# Low-Dose Aspirin in High-Risk Individuals With Screen-Detected Subsolid Lung Nodules: A Randomized Phase II Trial

**DOI:** 10.1093/jncics/pkaa096

**Published:** 2020-10-20

**Authors:** Bernardo Bonanni, Davide Serrano, Patrick Maisonneuve, Giulia Veronesi, Harriet Johansson, Valentina Aristarco, Clara Varricchio, Massimiliano Cazzaniga, Matteo Lazzeroni, Cristiano Rampinelli, Massimo Bellomi, Manuela Vecchi, Lorenzo Spaggiari, Lana Vornik, Powel H Brown, Therese Beavers, Aliana Guerrieri-Gonzaga, Eva Szabo

**Affiliations:** 1 Division of Cancer Prevention and Genetics, IEO European Institute of Oncology IRCCS, Milan, Italy; 2 Division of Epidemiology and Biostatistics, IEO European Institute of Oncology IRCCS, Milan, Italy; 3 Division of Thoracic Surgery, IRCCS San Raffaele Scientific Institute, Milan, Italy; 4 Faculty of Medicine and Surgery, Vita - Salute, San Raffaele University, Milan, Italy; 5 Department of Medical Imaging and Radiation Sciences, IEO European Institute of Oncology IRCSS, Milan, Italy; 6 Department of Oncology and Hemato-oncology, University of Milan, Milan, Italy; 7 IEO European Institute of Oncology IRCCS, Milan, Italy; 8 IFOM, Fondazione Istituto FIRC di Oncologia Molecolare, Cogentech S.R.L. Benefit Corporation with a Sole Shareholder, Milan, Italy; 9 Division of Thoracic Surgery, IEO European Institute of Oncology IRCCS, Milan, Italy; 10 Department of Clinical Cancer Prevention, The University of Texas MD Anderson Cancer Center, Houston, TX, USA; 11 Division of Cancer Prevention, National Cancer Institute, Bethesda, MD, USA

## Abstract

Lung cancer screening by helical low-dose computed tomography detects nonsolid nodules that may be lung adenocarcinoma precursors. Aspirin’s anti-inflammatory properties make it an attractive target for prevention of multiple cancers, including lung cancer. Therefore, we conducted a phase IIb trial (NCT02169271) to study the efficacy of low-dose aspirin to reduce the size of subsolid lung nodules (SSNs). A total of 98 current or former smokers (67.3% current) undergoing annual low-dose computed tomography screening with persistent SSNs were randomly assigned to receive aspirin 100 mg/day or placebo for 1 year. There was no difference in change in the sum of the longest diameters of target nodules in the placebo and aspirin arm after 12 months of treatment (-0.12 mm [SD = 1.55 mm] and +0.30 mm [SD= 2.54 mm], respectively; 2-sided *P* = .33 primary endpoint). There were no changes observed in subgroup analyses by individual characteristics or nodule type. One year of low-dose aspirin did not show any effect on lung SSNs. SSNs regression may not be the proper target for aspirin, and/or longer duration may be needed to see SSNs modifications.

Data from randomized clinical trials evaluating aspirin efficacy to prevent cardiovascular diseases have supported the role of aspirin as a cancer prevention agent (1). Further analyses have highlighted the potential of reduced cancer mortality with 5 years or more of aspirin treatment and 20 years follow-up: aspirin treatment was associated with a 35% mortality reduction in overall gastrointestinal cancers and nearly a 30% reduction in lung cancer mortality (*P *=* *.002) (2). Seeing an effect on mortality at 5 years, a preventive activity on premalignant lesions might be detectable already at 1 year. 

Helical low-dose computed tomography (ld-CT) screening can detect early-stage lung cancers, resulting in a 20% decrease in lung cancer mortality (3,4). Within CT screening programs, noncalcified nodules have been shown to be associated with increased cancer risk. In particular, subsolid (nonsolid or partially solid) lung nodules (SSNs) may represent adenocarcinoma precursors and are associated with increased long-term lung cancer risk (5). Focusing on persistent SSNs detected with ld-CT, a phase IIb randomized study was performed to assess aspirin’s effect on nodule size and other surrogate biomarkers.

Eligible participants were aged 50 years or older who were undergoing annual ld-CT screening at the European Institute of Oncology (Milan, Italy; COSMOS 1 and 2 trials) (6) or the MD Anderson Cancer Center (Houston, TX). Participants had a smoking history of 20 or more pack-years (current or former smokers; smoking cessation <20 years) and SSNs detected with ld-CT. The target nodule(s) had to be persistent (at least for 3 months) with a size between 4 mm and 10 mm and, if larger than 10 mm, with volume doubling time greater than 400 days. The main exclusion criteria were chronic treatment (at least twice per week for more than 3 months) with nonsteroidal anti-inflammatory drugs and history of allergic reactions to them. The study was approved by the European Institute of Oncology review board (https://clinicaltrials.gov/ct2/show/NCT02169271), and all the participants signed the informed consent.

Participants were randomly assigned to receive aspirin 100 mg/d or placebo for 12 months in a double-blind fashion. Complete physical exam, blood and urine collections for safety, and biomarker measurements (see the Supplementary Methods and Supplementary Table 1, available online) were performed at baseline, 6 months, and 12 months.

Lung nodules were measured with ld-CT at baseline and after 12 months as previously described (6) (see the Supplementary Methods, available online). To minimize the imbalance in treatment arms, a stratified blocked randomization strategy considering screening center, sex, smoking habits, and nodule type (nonsolid vs partially solid) was used.

Distributions of continuous variables were presented by the mean and standard deviation (SD), and comparison between treatment arms was assessed by the student *t* test. Differences in the distribution of dichotomous or categorical variables were assessed using the χ^2^ test or the Fisher exact test when the number of subjects in a cell was small. Per-nodule analyses were performed using random effects (mixed) linear models. Change in the sum of the longest diameters of the baseline target nodules between baseline and 12 months and their absolute differences were plotted using a waterfall plot for each single subject. All analyses were performed with SAS version 9.4 (Cary, NC). All *P* values are 2-sided, and *P*  values less than .05 are considered statistically significant.

The original sample size of 128 patients (64 per arm) was calculated assuming a -0.2 mm average reduction of the sum of the longest diameter of all SSNs at 12 months with respect to baseline in the placebo arm [data derived from prior similar clinical trial (7)] vs an expected average reduction of -1.0 mm in the aspirin group, with estimated effect size of 0.5. The study was stopped because of low accrual after random assignment of 98 individuals and thus had 80% power to detect an average reduction of -1.1 mm in the aspirin group (effect size = 0.56) or 69% power to detect the anticipated average reduction of -1.0 mm in the aspirin group. More endpoints and statistical analyses are described in the Supplementary Materials (available online).

Between December 2014 and June 2017, 619 ld-CT scans showing potentially eligible nodules were reviewed. A total of 109 individuals signed written informed consent and underwent baseline visit, and 98 were randomly assigned (consort diagram, Figure 1). The most frequent reasons for noneligibility were the nonpersistence of the target nodule (38.0%) and participant comorbidity or use of prohibited medications (35.9%).

**Figure 1.**
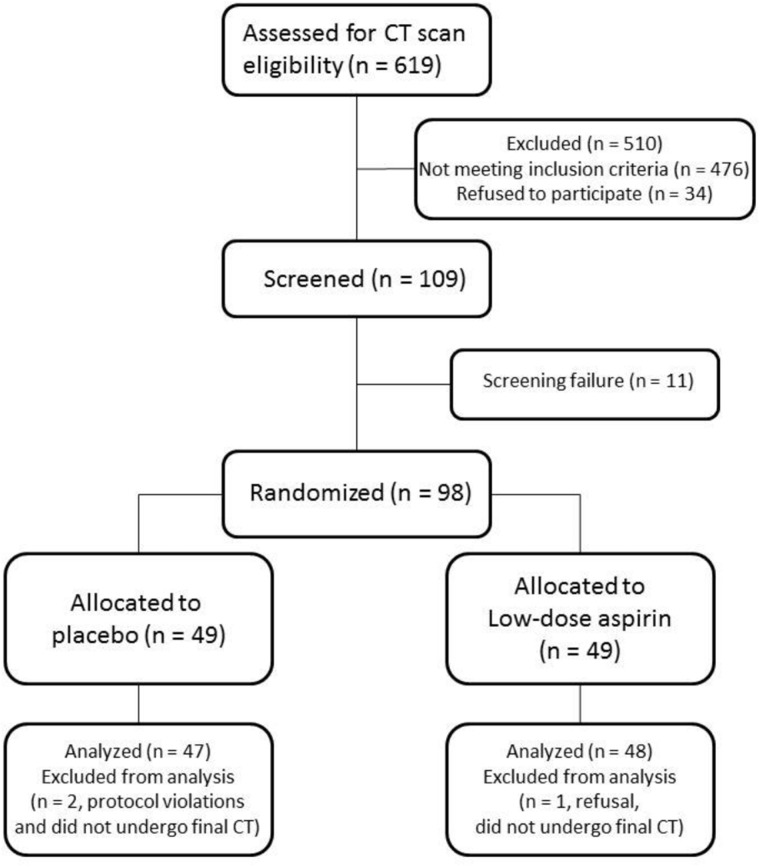
Trial flow diagram. CT = computed tomography.

The participants’ characteristics are shown in Supplementary Table 1 (available online); 43.8% were male, mean age was 64.6 years, and 67.3% were current smokers. Self-reported smoking status was consistent with urinary cotinine levels (data not shown). Compliance was good as indicated by the lower thromboxane B2 and prostaglandin E metabilite (PGEM) levels in the aspirin arm (Supplementary Table 2, available online).

The primary endpoint was the difference of the sum of longest diameters of SSNs in a person-specific analysis. The results showed no change after 12 months of treatment (placebo -0.12 mm [SD = 1.55 mm] vs aspirin 0.30 mm [SD = 2.54 mm]; *P *=* *.33) (Table 1 and Figure 2). Table 1 also shows secondary analysis on SSNs, using modified response evaluation criteria in solid tumors, which took into account all the nodules and characterized the responses as complete, partial, stable, or progressive (*P *=* *.44) (8). Other subgroups analyses per sex, smoking status, type of nodules, or body mass index (<25 vs ≥25 kg/m^2^) did not modify the results. Furthermore, a per-nodule analysis (volume and density variables) did not show any difference between study arms (*P *=* *.99 and *P *=* *.64, respectively).

**Figure 2.**
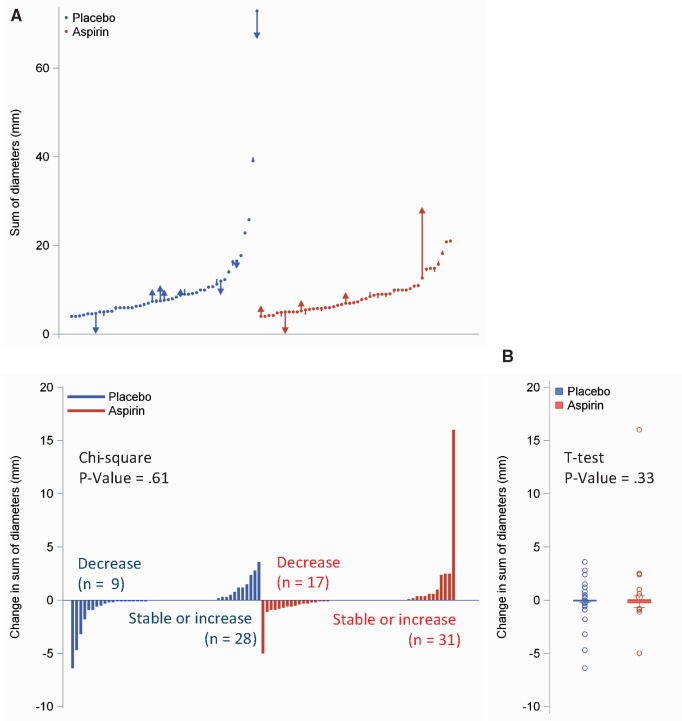
Change in target nodules size. Panel **(A)**: Waterfall plot showing the change in the sum of the longest diameters of baseline target nodules per subject in the placebo arm and in the aspirin arm. Each point on the *x*-axis represents a distinct individual. **Arrows** indicate increase or decrease of the sum of diameters from baseline. Panel **(B)**: Means (SD) of the sum of the longest diameters of baseline target nodules are shown.

**Table 1.** Primary and secondary outcomes

Outcomes	Placebo (n = 49)	Aspirin (n = 49)	*P*
Person-specific analysis			
Sum of longest diameters of baseline target nodules, mean (SD), mm			
Size at baseline	11.0 (11.3)	8.5 (4.3)	.16^a^
Size at 12 months^b^	10.7 (10.6)	8.9 (5.2)	.31^a^
Size difference (12 months^b^ baseline)	−0.12 (1.55)	+0.30 (2.54)	.33^a^
Subgroup analysis of size difference (12 months baseline)			
Among males (n = 43)	+0.05 (1.84)	+0.67 (3.84)	.52^a^
Among females (n = 55)	−0.25 (1.33)	+0.04 (0.81)	.35^a^
Among current smokers (n = 66)	−0.21 (1.24)	+0.38 (3.08)	.32^a^
Among former smokers (n = 32)	+0.06 (2.07)	+0.13 (0.74)	.89 ^a^
Among individuals with nonsolid nodules (n = 52)	−0.29 (1.74)	+0.32 (3.40)	.42^a^
Among individuals with partially solid nodules (n = 46)	+0.09 (1.30)	+0.27 (0.82)	.59^a^
Change in the sum of longest diameters of baseline target nodules,^b^ No. (%)			
Stable or increase (difference ≥0)	28 (59.6)	31 (64.6)	—
Decrease (difference <0)	19 (40.4)	17 (35.4)	.60^c^
Modified RECIST^b^ (n = 95), No. (%)			
Complete or partial response	2 (4.3)	1 (2.1)	—
Stable disease	40 (85.1)	37 (77.1)	—
Progression of disease	5 (10.6)	10 (20.8)	.44^d^
Nodule-specific analysis			
Sum of diameters of single baseline target nodules (n = 132), mean (SD), mm			
Size at baseline	7.2 (2.7)	6.8 (2.8)	.50^e^
Size at 12 months	7.1 (2.9)	6.9 (4.2)	.77^e^
Size difference (12 months baseline)	−0.1 (1.1)	0.1 (2.4)	.50^e^
Nodule volume (n = 131), mean (SD), mm^3^			
Volume at baseline	151 (151)	138 (139)	.35^e^
Volume at 12 months	147 (159)	134 (175)	.66^e^
Volume difference (12 months baseline)	−4.8 (134)	−4.5 (78)	.99^e^
Nodule density (n = 129), Hounsfield unit, mean (SD)			
Density at baseline	−663 (98)	−628 (76)	.23^e^
Density at 12 months	−636 (113)	−604 (99)	.33^e^
Density difference (12 months baseline)	28 (78)	24 (65)	.64^e^

a *P* value was calculated using a 2-sided *t* test. RECIST = response evaluation criteria in solid tumors.

b Missing for 3 subjects (2 placebo, 1 aspirin).

c *P* value was calculated using a 2-sided χ^2^ test.

d Fisher exact test was used to calculate 2-sided *P* values.

e Mixed-model *P* value.

Reported side effects were consistent with aspirin’s known profile (Supplementary Table 3, available online), with a statistically significant increase in minor hemorrhagic events reported with aspirin (*P *=* *.03). Notably, 4 lung cancer cases were diagnosed, 2 in each arm.

Aspirin's clinical benefit in cancer prevention is seen after a prolonged treatment. Our current study showed no effect on SSNs after 1 year of treatment. Potential reasons for these results include the limited duration of treatment, the fact that SSNs may be an off-target intermediate endpoint for aspirin (most SSNs do not progress quickly and not all are cancer precursors), and the possibility that aspirin prevents metastasis rather than progression of preneoplasia. Dose can be another issue; Rothwell and colleagues’ data reported no difference between low or high doses (2), but recently they showed that aspirin may lose activity in higher body mass index subjects (9). Our data showed no difference between normal weight and overweight or obese participants.

The antitumor effects of aspirin have been linked to reduced prostaglandin production via the inhibition of cyclooxygenase enzymatic activities (10). Sequential metabolism of arachidonic acid leads to the production of downstream prostaglandins and thromboxanes. Inhibition of serum thromboxane B2 and urinary PGEM in the aspirin arm may indicate the potential preventive activity. Both prostanoids modulate cell proliferation, apoptosis, and invasion (10). One more reason for this null result could be that the aspirin preventive effects act through these pathways at a very early stage of carcinogenesis, as has been suggested for colorectal cancer (11), before the appearance of a preneoplastic lesion. Whereas this evidence is quite strong for colorectal cancer, the clinical impact on lung cancer development has to be proven.

In conclusion, 1 year of low-dose aspirin was not able to reduce the size of SSNs, a non–small cell lung cancer risk marker. Our trial was too small and of too short a duration to assess the effect of aspirin on cancer development. Identification of appropriate intermediate endpoints to assess preliminary efficacy in lung cancer prevention trials remains challenging.

## Funding

National Cancer Institute Contract No. HHSN261201200034I

## Notes

**Role of the funder:** The funder had a role in the study design, data interpretation, and manuscript writing. The funder had no role in the collection or analysis. The decision to submit the manuscript for publication has been shared between the principal investigator and the funder. 

**Disclosures:** Veronese discloses, outside the topic of the study, personal fee from ab medica, Medtronic, and Johnson & Johnson. The other authors have no conflicts of interest to disclose.

**Author contributions:** BB: Study design and manuscript preparation; DS: Study design and manuscript preparation; PM: Study design, statistical analysis, and manuscript preparation; GV: Study design and patients recruitment; HJ: Laboratory analyses; VA: Laboratory analyses; MV: Laboratory analysies; CV: Patients recruitment; MC: Patients recruitment; ML: Study design and patients recruitment; CR: Lung nodules revision; MB: Patients recruitment; PPDF: Laboratory analyses; LS: Patients recruitment; LV: Administrative director; PHB: Study design; TB: Patients recruitment; AGG: Study design coordination and manuscript preparation; ES: Study design and manuscript preparation. 

**Acknowledgments:** Authors thank Bayer AG for kindly providing the drug and placebo free of charge.

This work was partially supported by: Italian Ministry of Health with “Ricerca corrente” and 5xmille funds and The University of Texas MD Anderson Cancer Center Duncan Family Institute for Cancer Prevention and Risk Assessment. We want to thank Eleonora Ancona, MD, and Marta Minotti, MD, for the support in screening and reviewing the CT scans and Raffaella Bertolotti, data manager of the COSMOS studies, and Micol Tillhon, PhD, for the analysis of circulating miRNA biomarkers.

## Data Availability

The raw data of this manuscript can be available upon reasonable request. As trial sponsored by National Cancer Institute, the request has to be made through the following website: https://cdas.cancer.gov/learn/eppt/browse/. The redacted study protocol and statistical analysis plan will also be shared upon request. 

## Supplementary Material

pkaa096_Supplementary_Data
