# Supplementary material for: Low-Dose Aspirin in High-Risk Individuals With Screen-Detected Subsolid Lung Nodules: A Randomized Phase II Trial
Source: JNCI Cancer Spectr. 2020 Oct 20;4(6):pkaa096. doi: 10.1093/jncics/pkaa096 (PMC7771428; doi:10.1093/jncics/pkaa096)
Supplement: pkaa096_Supplementary_Data [file pkaa096_supplementary_data.pdf]

Supplementary Methods

Morning fasting blood and urine samples were collected for biomarker analysis. Serum was separated (blood were left clotting at room temperature for 30-60 minutes than spun at 3000 rpm (1000 g, Megafuge 2.0 – Heraeus), and both serum and urine samples were stored at -80°C until analysis.

Serum thromboxane B2 (TXB2; the stable metabolite for thromboxane A2) was measured by a competitive enzyme linked immunosorbent assay (R&D Systems Inc., Minneapolis, MI). High sensitivity C-reactive protein was measured with turbidimetric method for Cobas Integra 800, Roche Diagnostics, Mannheim, Germany. Urinary concentrations of PGEM, a prostaglandin E2 metabolite that is a product of the cyclooxygenase pathway, were measured by a competitive enzyme immunoassay kit (Cayman Chemical Company, Ann Arbor, MI), and urinary leukotriene E4 were measured by a competitive enzyme immunoassay kit (Cayman Chemical Co., Ann Arbor, Michigan, USA). To measure tobacco product exposure, NicAlert® (Nymox Pharmaceutical Corporation, Saint Laurent, QC), a semi-quantitative immunochromatographic assay for urinary cotinine measurements, was used. Serum miRNA purification and expression profiling were performed according to a previously developed protocol, a 34 microRNA signature to identify patients with early stage non-small cell lung carcinomas (1).

Helical low-dose CT measurements: longest nodule diameters were measured by a dedicated radiologist while volumes and volume doubling time have been automatically calculated by Lung VCAR specific software (GE healthcare). Lung nodule maximum and mean density has been measured by the lung VCAR (GE) software before and after treatment (2).

REFERENCE

1. Bianchi F, Nicassio F, Marzi M et al. A serum circulating miRNA diagnostic test to identify asymptomatic high-risk individuals with early stage lung cancer. *EMBO Mol Med.* 2011;3(8):495-503.

2. Yankelevitz DF, Reeves AP, Kostis WJ, Zhao B, and Henschke CI. Small pulmonary nodules: volumetrically determined growth rates based on CT evaluation. *Radiology.* 2000;217(1):251-256.

Supplementary Table 1. Characteristics of participants at baseline

	Placebo N (%)	Aspirin N (%)
Total	49 (100.0)	49 (100.0)
Screening Program		
COSMOS-1	19 (38.8)	19 (38.8)
COSMOS-2	30 (61.2)	29 (59.2)
MD Anderson	0 (0.0)	1 (2.0)
Gender		
Male	22 (44.9)	21 (42.9)
Female	27 (55.1)	28 (57.1)
Age group		
55-59	5 (10.2)	5 (10.2)
60-64	19 (38.8)	20 (40.8)
65-69	18 (36.7)	19 (38.8)
70+	7 (14.3)	5 (10.2)
Smoking status		
Current smoker	33 (67.3)	33 (67.3)
Former smoker	16 (32.7)	16 (32.7)
Smoking duration (years)		
Mean ± SD	42.9 ± 8.1	44.8 ± 7.0
[range]	[25-56]	[29-64]
Smoking cessation (years)		
Mean ± SD	7.8 ± 5.8	6.6 ± 5.5
[range]	[0-19]	[0-16]
Pack-years		
Mean ± SD	41.2 ± 17.1	44.6 ± 19.8
[range]	[8-80]	[15-96]
Body mass index		
Normal	29 (59.2)	24 (49.0)
Overweight	14 (28.6)	15 (30.6)
Obese	5 (10.2)	8 (16.3)
Missing	1 (2.0)	2 (4.1)
Nodules type		
Non solid	26 (53.1)	26 (53.1)
Partially solid	23 (46.9)	23 (46.9)
Target nodules number		
1	38 (77.6)	40 (81.6)
2	5 (10.2)	5 (10.2)
3	4 (8.2)	4 (8.2)
4+	2 (4.1)	0 (0.0)
Non-target nodules number		
0	28 (57.1)	26 (53.1)
1	13 (26.5)	15 (30.6)
2	4 (8.2)	5 (10.2)
3	3 (6.1)	0 (0.0)
4+	1 (2.0)	3 (6.1)

Supplementary Table 2. Secondary outcomes: Modulation of circulating biomarkers

	Placebo	Aspirin	P-values
Thromboxane B2 (ng/mL)			
Baseline (n=94)	61.1 ± 37.3	60.4 ± 32.6	0.41
12 months (n=94)	81.6 ± 63.3	24.6 ± 45.8	<0.0001
Difference (n=94)	+20.5 ± 50.7	-35.8 ± 53.4	<0.0001
Prostaglandin E metabolites[a]			
Baseline (n=92)	334 ± 213	385 ± 218	0.26
12 months (n=92)	364 ± 222	324 ± 216	0.39
Difference (n=92)	+29.8 ± 215	-60.7 ± 210	0.04
Leukotriene E4[a]			
Baseline (n=92)	1226 ± 909	1701 ± 1670	0.09
12 months (n=92)	1217 ± 567	1466 ± 1126	0.19
Difference (n=92)	-9 ± 592	-235 ± 1240	0.27
High sensitive C-reactive protein (mg/dL)			
Baseline (n=94)	0.29 ± 0.52	0.47 ± 1.01	0.27
12 months (n=94)	0.20 ± 0.13	0.35 ± 0.42	0.03
Difference (n=94)	-0.08 ± 0.50	-0.12 ± 0.88	0.80
miRNA prediction risk score			
Baseline (n=94)	10.0 ± 10.6	10.4 ± 9.5	0.86
12 months (n=94)	10.4 ± 8.7	10.3 ± 8.7	0.95
Difference (n=94)	+0.36 ± 7.36	-0.14 ± 8.94	0.77

[a] normalized to urinary creatinine concentration;

12-month PGEM and Leukotriene E4 were not available for 6 subjects; 12-month HS-CRP, TXB2 and miRNA score were missing for 4 subjects.

After exclusion of non-compliant subjects (ie. Subjects allocated to aspirin who did not show a modulation of TXB2), the association with the modulation of PGEM remained virtually unchanged (mean difference in the aspirin group =-65.0±207, P=0.05).

Supplementary Table 3. Adverse events and serious adverse events

	Placebo	Aspirin	P-value
Number of AEs possibly or probably related to treatment	19 AEs in 10 patients	30 AEs in 12 patients	
Grade of AEs			
Mild	13	17	
Moderate	6	11	
Severe	0	2	0.68
AE reported at least once per patient			
Dyspepsia	1	4	0.36
Gastroesophageal reflux disease	2	0	1.00
Gastritis	1	0	1.00
Gastrointestinal/abdominal pain/colitis	5	5	1.00
Diarrhea	-	1	1.00
Hemorrhoids	1	1	1.00
Oral hemorrhage	-	1	1.00
Epistaxis	-	2	0.49
Lower gastrointestinal hemorrhage	-	1	1.00
Hemorrhoidal hemorrhage	1	2	1.00
Hematuria	-	1	1.00
Hematoma	-	2	0.49
Eye disorders - Other, specify	-	1	1.00
Vascular disorders - Other, specify	1	-	1.00
Any kind of hemorrhage/hematoma	1	8	0.03
Any Other AE	10	8	0.79
Number of AEs unrelated or unlikely related to treatment	175 AEs in 39 patients	167 AEs in 40 patients	
Serious Adverse Event			
Lung cancers (+ suspected)	2[a](+1)	2	1.00
Bladder cancer	0	2[b]	0.49
Liposarcoma	0	1	1.00
Hemorrhoids	0	1	1.00
Anal rhagades	0	1	1.00
Penile implant	1	0	1.00
Circumcision	0	1	1.00

[a] 1 lung cancer was present at baseline (Protocol violation) and was not included in the primary outcome analysis as 12-month data were not available.

[b] 2 patients developed bladder cancer and 1 of them had a subsequent recurrence.
